# General Pathology, or the Science of the Causes, Nature, and Course of the Pathological Disturbances Which Occur in the Living Subject

**Published:** 1900-01

**Authors:** 


### Book Reviews.

General Pathology, or the Science of the Causes, Nature, and Course of the Pathological Disturbances which Occur in the Living Subject. By Dr. Ernst Ziegler, Professor of Pathological Anatomy and of General Pathology at the University of Freiburg, in Breslau. New York: William Wood & Co. 1899.

The American translation of the ninth revised German edition of Ziegler's pathology is before the profession and is a magnificent work from every point of view. Under the able editorship of Dr. Albert H. Buck, assisted by a competent corps of translators—each in himself an expert pathologist—a work has been brought forth that must maintain the high standard of excellence that Ziegler holds in America. The temptation has been great to substitute a compend of pathology for the classical text-book, which has so long been such a valued addition to the student's and physician's library, but happily Professor Ziegler did not yield and medicine has been enriched thereby. Many important changes were made in the eighth edition, chapters rewritten, new illustrations added and important scientific investigations and researches incorporated. The changes in the ninth edition are not so numerous, but yet important. The chapters on retrograde disturbances of nutrition and infiltrations of the tissues, and that on tumors have been entirely rewritten. In the former, the different kinds of degeneration which lead to the formation of hyaline products are divided into four groups, giving good or general ideas of the different processes under consideration. The chapter on pigmentation has been augmented by some general remarks on the pathological absence of pigment, in which the author takes up for consideration albinism, vertigo and leukoderma.

Chapter VII. on tumors has also been largely rewritten. Ziegler now divides tumors into three large groups: (1) tumors of the connective tissue; (2) epithelial tumors; and (3) teratoid tumors and cysts. The epithelial tumors have been divided into two lesser groups, in one of which the papillary epitheliomata, the adenomata and the cystoadenomata are to be placed, while in the other belong the carcinomata and the cystocarcinomata. The author still holds to his former theory regarding the nature and origin of tumors, notwithstanding the many attacks made upon him. Ziegler establishes and groups the tumors according to origin: (1) those which arise from some localised predisposition of the tissues of a distinctively congenital nature; (2) a second group is developed after traumatic injuries of the tissues. In a third group of cases the development of the new growth follows an inflammation, especially if accompanied by ulceration and the formation of a scar. The tumors of the fourth group seem to owe their development to the unequal atrophy of the elements which make up a tissue, as a result of which certain opposing forces are removed or lessened. Our knowledge of the cause of tumors up to the present time may be thus summed up: hereditary and acquired conditions of certain cells and cell groups, which express themselves in a tendency to increased formative activity and to the production of atypical tissue lead to the formation of tumors. Ziegler is decidedly not of the opinion that cancer is a parasitic disease. Thus, he says, in the few cases in which genuine parasites have been found in the tissues, they may perfectly well have entered after the tumor had begun to develop. Under such circumstances they can in no sense be looked upon as the cause of the development of the carcinoma. Of the many so-called parasites described, he says they are nothing but degenerated nuclei and karykinoketic figures, or leucocytes (or the products of their destruction) which have been included in tumor cells or products of the cell-protoplasm, especially keratohyalin and colloid. Ziegler further states that "there are three facts which mitigate against the idea of a parasitic origin for these tumors. First, no parasites can be demonstrated in the beginning of the cancer development; second, the whole course of the disease is quite different from that of the epithelial growth produced by parasites, and, third, the formation of metastatic tumors is due, beyond all question, to the transportation of cancer cells." These views of this eminent and thorough pathologist are somewhat contrary to the view accepted by many other eminent and thorough pathologists, that cancer is a parasitic disease. Fortunately, the controversy may soon be settled by the definite announcement of the discovery of the microörganism of cancer, or quelled for the time being by failure to detect a parasitic growth.

Chapter VIII., on disturbances of development and the resulting malformation, is an interesting section and fully illustrated. Chapter IX. deals with fission fungi which exist as parasites and the diseases caused by them. Chapter X. treats of the mould and yeast fungi, and Chapter XI. treats of the various parasites which infest the human system. A very complete general index adds much to the value of the work. The illustrations, 544 in number, many in colors, some full-page colored plates, are all excellent, well chosen, finely executed and well described. As a text-book and work of reference this ninth edition will maintain its undisputed claim to professional favor and popularity. W. C. K.

